# Factors determining antenatal care utilization among mothers of deceased perinates in Ethiopia

**DOI:** 10.3389/fmed.2023.1203758

**Published:** 2023-11-08

**Authors:** Neamin Tesfay, Mandefro Kebede, Negga Asamene, Muse Tadesse, Dumesa Begna, Fitsum Woldeyohannes

**Affiliations:** ^1^Center of Public Emergency Management, Ethiopian Public Health Institute, Addis Ababa, Ethiopia; ^2^Health Financing Program, Clinton Health Access Initiative, Addis Ababa, Ethiopia

**Keywords:** antenatal care, count modeling, perinatal death surveillance, pastoralist regions, Ethiopia

## Abstract

**Introduction:**

Receiving adequate antenatal care (ANC) had an integral role in improving maternal and child health outcomes. However, several factors influence the utilization of ANC from the individual level up to the community level factors. Thus, this study aims to investigate factors that determine ANC service utilization among mothers of deceased perinate using the proper count regression model.

**Method:**

Secondary data analysis was performed on perinatal death surveillance data. A total of 3,814 mothers of deceased perinates were included in this study. Hurdle Poisson regression with a random intercept at both count-and zero-part (MHPR.ERE) model was selected as a best-fitted model. The result of the model was presented in two ways, the first part of the count segment of the model was presented using the incidence rate ratio (IRR), while the zero parts of the model utilized the adjusted odds ratio (AOR).

**Result:**

This study revealed that 33.0% of mothers of deceased perinates had four ANC visits. Being in advanced maternal age [IRR = 1.03; 95CI: (1.01–1.09)], attending primary level education [IRR = 1.08; 95 CI: (1.02–1.15)], having an advanced education (secondary and above) [IRR = 1.14; 95 CI: (1.07–1.21)] and being resident of a city administration [IRR = 1.17; 95 CI: (1.05–1.31)] were associated with a significantly higher frequency of ANC visits. On the other hand, women with secondary and above education [AOR = 0.37; 95CI: (0.26–0.53)] and women who live in urban areas [AOR = 0.42; 95 CI: (0.33–0.54)] were less likely to have unbooked ANC visit, while women who resided in pastoralist regions [AOR = 2.63; 95 CI: (1.02–6.81)] were more likely to have no ANC visit.

**Conclusion:**

The uptake of ANC service among mothers having a deceased perinate was determined by both individual (maternal age and educational status) and community (residence and type of region) level factors. Thus, a concerted effort is needed to improve community awareness through various means of communication by targeting younger women. Furthermore, efforts should be intensified to narrow down inequalities observed in ANC service provision due to the residence of the mothers by availing necessary personnel and improving the accessibility of service in rural areas.

## Introduction

Antenatal care (ANC) is routine medical and nursing care recommended as a precautionary measure for pregnant women to prevent, detect, and treat any pregnancy-related complications (1). ANC service is also considered one of the pathways to a continuum of care, used to identify high-risk pregnancies and take appropriate intervention on the spot (2, 3). Globally, 87% of women had access to ANC service by trained health personnel at least once; however, only two in three (65%) received at least four antenatal visits. The coverage became further lower in Sub-Saharan Africa and South Asian countries with a proportion of 52 and 48%, respectively (4).

Ethiopia has made remarkable progress in improving the coverage of ANC services in the last two decades (5). The coverage has increased from 28% in 2005 to 74% in 2019 (6). In line with this, the number of women who attended four and more ANC visits (ANC 4+) has also increased from 12% in 2005 to 43% in 2019 (7).

Effective utilization of ANC has a positive role in improving maternal and child health in addition to ameliorating health promotion (8). Reducing perinatal death, averting preterm delivery, and diminishing the rate of having a low-birth-weight baby are some benefits of ANC in maintaining child health while lowering the chance of having maternal near and postpartum hemorrhage listed under maternal health (9–16). Moreover, enhancing institutional delivery and encouraging the utilization of postpartum contraceptives are the major benefit of ANC among others included under the category of boosting health service utilization (17, 18).

To maximize the benefit of ANC visits, different measures were taken per the recommendation of the World Health Organization (WHO), one of the recommendations was the introduction of a minimum of eight visits along with an early (i.e., first-trimester) initiation of the visit (19). Accordingly, Ethiopia has accepted the recommendations and has made slight progress on its implementation, where only 15% of women had eight and more visits, and 28% of pregnant women-initiated ANC visits within the first trimester (6, 20).

Ethiopia has also taken various interventions to improve the coverage and quality of the ANC service by improving community engagement through health extension programs, community-based health insurance (CBHI), and forming an organized community health structure (21, 22). On the other hand, the country has made a stride in ensuring sustainable financing by establishing a healthcare financing system (23). On top of all the above-mentioned measures, the country has established maternal and perinatal death surveillance and response (MPDSR) system by acknowledging the gaps in the availability of robust information for decision-making (24, 25). ANC service utilization was included in the surveillance system to evaluate and review maternal and perinatal death through the chain of events taking place during the continuum of care (26).

Despite taking these measures, the country had not achieved the target set in 2020. The target was to improve the coverage of more than four ANC visits to 68% within 5 years of implementation from 2016 to 2020 (27). The plan was not successful due to the presence of noticeable regional variation and the lack of an effective monitoring mechanism for the proposed intervention (28–31). Maximizing and maintaining the quality of service provided during the ANC visit was also a major challenge that come across with coverage of the service (32, 33). Ethiopia has made some tangible strides in improving the provision of the essential element of ANC services such as blood pressure measurement, nutritional counseling, blood, and urine sample examination…etc. (6). However, the quality-of-service provision is still being challenged by a lack of trained personnel and essential equipment, along with the poor documentation practice at the health facilities (34–36). In addition to the palpable gaps in the quality of the service, the service utilization remains unsatisfactory (37).

The ANC service utilization is determined by numerous factors related to the mother, and the health facility (38–40). Level of education, wealth index, maternal parity, maternal age, spousal support, employment status, ethnicity, religion, pregnancy intention, media exposure, and decision-making power are some of the maternal (individual) level factors that determined the utilization of ANC service (41–48), while the type of region, proximity to a health facility, presence of respectful care, residence, enrollment of health insurance are some of the facility(community) level factors (49–53).

Several studies have been conducted to pinpoint factors that affect the utilization of ANC services in Ethiopia. However, most of these studies were limited to small geographical areas, which may have had little influence on policy development and change (54–62). Furthermore, several studies failed to adopt the relevant counting model and instead relied on conventional methods of analysis, which have limitations in providing sufficient detail on a pattern of multiple ANC visits (43, 63–67). Some studies attempted to use the count model to determine the number of ANC visits using single-level regression (68–71); however, they failed to acknowledge the hierarchical nature of their dataset in the development model. Additionally, few studies considered the hierarchical nature of their data and did not consider overdispersion and excess zeros in their model development (72, 73). Moreover, most studies exclusively focused on pregnant women, overlooking other important segments of the study population. Furthermore, the findings of these studies were not well-integrated with national-level studies and policies, impeding further improvement and implementation of additional measures. Considering these overt gaps in terms of geographical coverage, methodological limitation, study population, and policy influence, this study attempt to address those aforementioned limitations and aims to identify factors associated with the utilization of ANC care among mothers of deceased perinate based on national surveillance data using an appropriate counting model.

## Method and materials

### Study setting

Ethiopia has an estimated population of 117,876,000 in 2021, out of which 17, 216,372 are under-five children (74). Administratively, Ethiopia has ten regions and two city administrations, namely Tigray, Afar, Amhara, Oromia, Somali, Benishangul-Gumuz, Southern Nations Nationalities, and Peoples Region (SNNPR), Sidama, Gambella, Harari, Addis Ababa city administration and Dire Dawa city administration (75). The country has high infant, under-five, and maternal mortality rates, (47 per 1000LBs), (59 per 1000LBs), and (412 per 100,00 LBs) respectively (5, 6).

### Data source and study participant

The study used data from Ethiopian Public Health Institutes (EPHI), which is collected and compiled from various health facilities across the country. It utilized an updated programmatical and epidemiological review of perinatal death data obtained from all perinatal death surveillance and response (PDSR) implementing regions for four consecutive years (2018–2021). The data was extracted through facility-based abstraction format (FBAF) and verbal autopsy (VA) (26). The source population for this study are all mothers who had a deceased perinate that was reviewed by the MPDSR committee during the study period. Accordingly, a total of 3,814 mothers of deceased perinates were included in the study. The PDSR data was hierarchical, i.e., the mothers of the deceased perinate were nested in 161 reporting health facilities and 45 provinces of the country.

### Study variables

#### Outcome variable

The number of ANC visits (non-negative integer) is the target response variable for which this study aims to identify a proper count regression model.

#### Explanatory variables

Several explanatory variables at the individual (woman), facility, and regional level were selected in consideration of recent literature findings related to ANC service utilization. Education status, maternal age, maternal parity, and religion were included under the individual-level (exclusive characteristics of individuals) factors that could affect the utilization of ANC; while residence, type of region, ownership of the health facility, and type of health facility were included under community-level factors (shared characteristics of the community).

The type of region was further classified into three categories (city administration, agrarian, and pastoralist) based on the cultural and socio-economic backgrounds of the population (76). Furthermore, the type of facility was codified into classes (primary, secondary, and tertiary facilities) according to their manpower, medical equipment, and type of service provision (77).

### Data management and statical analysis

Originally the data was captured in Epi-info version 7.2, and for data cleaning and further analysis, the data was exported to R version 4.2.1. Both descriptive [count, median ( $\tilde{x}$ ), and Chi-Square(X^2^)] and analytical analysis (Hurdle mixed Poisson model with mixed effects in the zero parts) were carried out and reported. Median was used as a measure of central tendency due to the skewness of the observations to the left (78).

#### Model building

A series of different count regression models were performed to select the best-fitted model, which is more suitable to the nature of the data. The step followed for data analysis are listed below.

##### Step one

Poisson single-level regression (PR) was carried out to assess the factors that determine the utilization of the ANC service among mothers of a deceased perinate. After the regression, the assumption of Poisson distribution was assessed, which operates under the presence of equal mean and variance (79).

##### Step two

The assumption of equidispersion (having equal mean and variance) was checked, and according to the finding the mean (x̄) was 2.55 with a variance (σ2) of 2.15. The x̄ is slightly higher than the σ2, which might indicate the presence of under dispersion (x̄ > σ2). In such a violation of the assumption of the PR model, negative binomial regression (NBR) is recommended (80). To have an objective measurement, the model adequacy was checked using Akaike’s Information Criterion (AIC) and Bayesian information criterion (BIC). As depicted in Table 1, the PR model has the smallest AIC and BIC than the NBR model.

**Table 1:** Model selection to analyze the number of antenatal care utilization among mothers of deceased perinate in Ethiopia,2021.

Name of model	AIC	BIC	lognormal
Poisson regression (PR)	13784.59	13872.04	−6878.295 (df = 14)
Negative binomial regression (NBR)	13786.64	13880.34	−6878.32 (df = 15)
Zero-inflated Poisson model (ZIPR)	13359.38	13534.28	−6651.691 (df = 28)
Hurdle Poisson regression (HPN)	13359.57	13534.47	6651.785 (df = 28)
Mixed Poisson regression (MPR)	13441.3	13522.5	−6707.648 (df = 13)
Zero-inflated Poisson mixed regression model (fixed effect) (MZIPR.fixed)	13241.17	13330.71	−6591.585 (df = 29)
Hurdle mixed Poisson regression model with only fixed effects (MHPR.fixed)	13318.44	13401.8	−6632.219 (df = 27)
Zero-inflated Poisson mixed model with mixed effects in the zero parts (MZIPR.ERE)	13013.74	13109.45	−6475.868 (df = 31)
Hurdle mixed Poisson model with mixed effects in the zero parts (MHPR.ERE)	12998.06	13081.43	−6472.031 (df = 27)

##### Step three

After confirmation of the PR model’s superiority over the NBR model, through the model adequacy test, the presence of excess zero in the model was assessed. The observed zero counts were 17.0%, which is higher than the expected 7.8% of zero counts. The discrepancy between the expected and observed zero counts lead to seeking a model that could handle the excess zero count (81, 82).

##### Step four

To handle the observed excess zero in regression, zero-inflated and hurdle regression (ZIR and HR) were adopted for the analysis of count data (83). The regression was performed using Poisson distribution through a model of ZIR and HR. Both models allow interpreting separate answers to the two questions (i) which factors influence whether a pregnant woman will attend ANC or not and (ii) which factors predict the number of times she will take ANC. A model adequacy test was performed, and Both models had similar model adequacy and quality in explaining the explanatory variables (Table 1). However, all the above-cited models (PR, NBR, ZIR, and HR) assumed that each observation was independent and identically distributed within the models, which violated the assumption of correlation with longitudinal and cluster data (84, 85).

##### Step five

A simple mixed Poisson regression model (MPR) was performed to handle the hierarchal nature of the data. Despite handling the clustering effect, the MPR model could not handle the excess zeros during the analysis (86). This drawback within the MPR model necessitates exploring another model.

##### Step six

To handle the clustering effect and excess zero counts with the data set, the zero-inflated Poisson mixed model fixed effect (MZIPR.fixed) and Hurdle mixed Poisson regression model with only fixed effects (MHPR.fixed) were considered to handle the limitation with MPR model. The model comparison was carried out using the likelihood ratio test score. The finding indicated the presence of room for further improvement of the model.

##### Step seven

Both MZIPR.fixed and MHPR.fixed models had a mixed effect on the count part. Thus, the implementation of the random effect in the zero parts was taken as part of further improvement for the model. A zero-inflated Poisson mixed regression model with mixed effects in the zero parts (MZIPR.ERE) and Hurdle mixed Poisson regression model with mixed effects in the zero parts (MHPR.ERE) were employed. As depicted in Table 1, MHPR.ERE was selected as the final best-fitted model due to its high likelihood ratio test score (87).

##### Step eight

The result of MHPR.ERE model was presented in two ways, the first part of the count segment of the model shows the effects of the considered factors on the frequency of ANC visits represented as an incidence rate ratio (IRR), while the second part of the zero-part model shows the effects of the considered factors on the women’s decision to take no ANC represented as adjusted odds ratio (AOR). Besides, the cluster effect was reported using variance and intra-cluster correlation (ICC) for both the zero and count parts of the model.

## Results

### Selected characteristics of the reported facility

**Figure 1:**
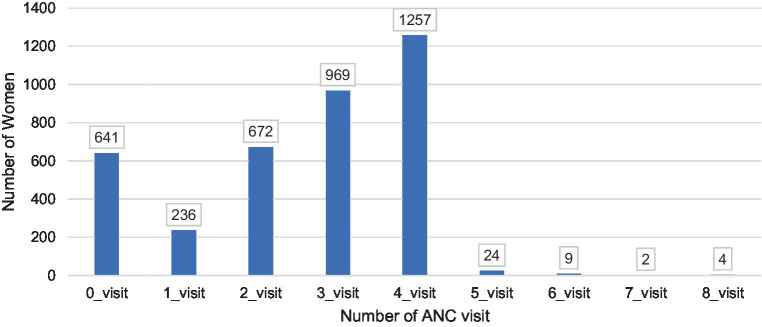
Distribution of the number of antenatal care (ANC) visits among mothers of deceased perinate in Ethiopia.

A total of 3,814 mothers of a deceased perinates were included in the study. As depicted in Figure 1, 33.0% of the women had four ANC visits while 25.1%, 17.8%, and 16.8% had three, two, and no visits, respectively. As shown in Table 2, the median varies significantly for the different background characteristics. The media of ANC visits was higher among women who visited secondary and tertiary health facilities (x̃ =4) than women who attend ANC visits within primary health care facilities (x̃ =3). Women who resided in Addis Ababa had a higher frequency of ANC visits (x̃ =4) as compared to women who reside in Gambella (x̃ =0) (Table 2).

**Table 2:** Median number of antenatal care (ANC) visits in Ethiopia according to reporting health facilities’ selected characteristics, and the associated statistical F-test and Chi-Square (*χ*^2^) test for equality of the means and medians, respectively.

Characteristics	Category	Frequency	Median (x̃)	F (*p*-value)	*χ*^2^ (*p*-value)
Ownership of the facility	Public	3,801	3	8.62 (0.000)	9.66 (0.000)
	Private	11	4		
	NGO	2	4		
Type of health facility	Primary	1999	3	49.15 (0.000)	125.99 (0.000)
	Secondary	879	4		
	Tertiary	936	4		
Source of data	Facility-based abstraction	3,639	3	1.08 (0.29)	1.79 (0.18)
	Verbal autopsy	175	3		
Reporting region	Addis Ababa	808	4	38.33 (0.000)	324.00 (0.000)
	Amhara	1,989	3		
	Benishangul-Gumuz	72	2		
	Dire Dawa	38	3		
	Gambella	4	0		
	Harari	21	2		
	Oromia	568	2		
	Sidama	154	2		
	SNNPR	96	3		
	Somali	64	0		
Year of reporting	2018	448	3	17.72 (0.000)	61.17 (0.000)
	2019	782	3		
	2020	879	3		
	2021	1705	3		
National	3,814		3		

### Selected characteristics of mothers of the deceased perinate

Women aged between 45 and 49 had a higher frequency of ANC visits (x̃ =4) than women aged between 40 and 44 (x̃ =3). Women who give birth in a health facility had a higher frequency of ANC visits (x̃ =3) than women who give birth at home and in transit (x̃ =2). Furthermore, women who resided in city administration had a higher frequency of ANC visits (x̃ =4) than women who resided in the pastoralist region (x̃ =1) (Table 3).

**Table 3:** Median number of antenatal care (ANC) visits in Ethiopia per the selected characteristics of mothers of the deceased perinate, and the associated statistical F-test and Chi-Square (χ^2^) test for equality of the means and medians, respectively.

Characteristics	Category	Frequency	Median (x̃)	F (*p*-value)	*χ*^2^ (*p*-value)
Maternal age	15–19	174	3	4.43 (0.000)	22.02 (0.000)
	20–24	954	3		
	25–29	1,444	3		
	30–34	737	3		
	35–39	413	3		
	40–44	80	3		
	45–49	12	4		
Maternal parity	0–1	413	3	2.51 (0.082)	6.06 (0.05)
	2_4	1,397	3		
	= > 5	2,004	3	30.95 (0.000)	53.85 (0.000)
Maternal religion	Christian	2,978	2		
	Muslim	812	2		
	Traditional	24	2		
Maternal education	Illiterate	2,168	3	66.27 (0.000)	135.57 (0.000)
	Primary	941	3		
	Secondary and above	705	3		
Place of delivery	Home	71	2	73.53 (0.000)	70.46 (0.000)
	On transit	265	2		
	Health facility	3,478	3		
Mode of delivery	Spontaneous vaginal delivery	3,071	3	14.09 (0.000)	55.88 (0.000)
	Operative vaginal delivery	520	3		
	Caesarian section	223	3		
Status of mother	Alive	3,353	3	15.44 (0.000)	19.78 (0.000)
	Died	461	3		
Residence	Rural	2,098	3	130.10 (0.000)	121.81 (0.000)
	Urban	1,716	3		
Type of region	Pastoralist	140	1	81.71 (0.000)	165.48 (0.000)
	City administration	867	4		
	Agrarian	2,807	3		

### Factors associated with the number of ANC visits

Per the results of the finally selected count-part model, as shown in Table 4, as maternal age increased by 1 year the frequency of ANC visits increased by 3% percent [IRR = 1.03; 95 CI: (1.01–1.09)]. The frequency of ANC visits is 8% [IRR = 1.08; 95 CI: (1.02–1.15)] higher among women with primary education, as compared to women with no education. Similarly, the frequency of ANC visits is 14% [IRR = 1.14; 95 CI: (1.07–1.21)] higher among women with secondary and above education as compared to women with no education. Furthermore, the frequency of ANC visits is 17% [IRR = 1.17; 95 CI: (1.05–1.31)] greater among women who resided in the city administration compared to women who resided in agrarian regions.

**Table 4:** Incidence rate ratio (IRR) of attending ANC visits and odds ratio (OR) of not attending any ANC visit with their 95% CI and *p*-values from the hurdle Poisson regression with a random intercept at both count-and zero-part (MHPR.ERE) models.

Characteristics	Categories	Count part (Number of ANC visits)	Zero part (No ANC attendance)
		IRR (95% CI)	AOR (95% CI)
Maternal age		1.03 (1.01–1.09) *	0.99 (0.98–1.01)
Educational status	No education	1	1
	Primary	1.08 (1.02–1.15) *	1.01 (0.78–1.31)
	Secondary and above	1.14 (1.07–1.21) ***	0.37 (0.26–0.53)***
Religion	Christian	1	1
	Muslim	1.03 (0.76–1.40)	1.33 (0.98–1.81)
	Traditional	1.02 (0.96–1.09)	2.07 (0.71–6.05)
Facility ownership	Public	1	1
	NGO	1.50 (0.69–3.25)	0.70 (0.23–2.43)
	Private	1.41 (0.97–2.03)	0.71 (0.04–13.18)
Residence	Rural	1	1
	Urban	1.04 (0.98–1.09)	0.42 (0.33–0.54)***
Type of region	Agrarian	1	1
	City admiration	1.17 (1.05–1.31) ***	1.27 (0.58–2.78)
	Pastoralist	0.89 (0.74–1.08)	2.63 (1.02–6.81)*
Random effect	$\mathrm{Variance}\;\left(\sigma^{2}\right)$	0.02	1.53
	ICC	0.01	0.31

*p < 0.05, **p < 0.001, ***p < 0.0001.

Regarding the zero parts of the model, the likelihood of not having an ANC visit decreased by 63% [AOR = 0.37; 95 CI: (0.26–0.53)] among women with secondary education than women with no education. Similarly, the likelihood of not having an ANC visit was reduced by 58% [AOR = 0.42; 95 CI: (0.33–0.54)] among women who resided in urban areas compared to women who resided in rural areas. In line with this, women who resided in pastoralist regions were nearly 3 times [AOR = 2.63; 95 CI: (1.02–6.81)] more likely to not attend ANC visits as compared to women who resided in agrarian regions. The estimated intra-cluster correlation (ICC) components in the count part (ICC = 0.01) and the zero parts (ICC =0.31) indicate significant province-level variation in the number of ANC visits, due to between-cluster heterogeneity (Table 4).

## Discussion

Per the final model selection criteria, hurdle Poisson regression with a random intercept at both count-and zero-part (MHPR.ERE) model was selected as the best-fitted model, by considering hierarchal and excess zero with the outcome variable. According to the final model, the utilization of ANC among mothers of a deceased perinate is determined by maternal age, educational status, residence, and type of region.

The final model output revealed that as maternal age increase by a year the frequency of ANC visits also increased among mothers of the deceased perinate. This finding was parallel with studies conducted in Ethiopia (Dire Dawa, Bench-Sheko, and East Wollega) (41, 47, 48), Afghanistan (42), Guinea (44), Ghana (88), and Congo (89). The plausible explanation may be linked to the level of awareness and knowledge related to ANC services being higher among older women due to previous experience of pregnancy (90). Moreover, adolescent pregnancy is always compounded by social ramifications, which makes a woman ashamed of their pregnancy. This results in heightened resistance to disclosing their pregnancy and seeking care timely (91). The explanation is also supported by evidence generated from the 2019 Ethiopian Demographic Health and Survey (EDHS), where only 36.4% of women below 20 years of age had more than four ANC visits, while the coverage was 45.7% among women between the age of 20 to 35 (6). In Ethiopia, not being in school, early marriage, non-use of contraceptives, and lack of open discussion with the parents on reproductive health issues had a high role in teenage pregnancy (92–94). Thus, the finding imply that a concerted effort is needed to improve awareness and address barriers to the utilization of ANC among younger women by providing youth friendly services.

The study also revealed that the education status of women was positively associated with the utilization of ANC services, i.e., educated women tend to attend more ANC services than those who are uneducated. The finding well agrees with studies conducted in Ethiopia (Jimma) (37), Nigeria (95), Mauritania (96), Benin (97), Ghana (51) Kenya (98), and Nepal (99). This could be explained by the role education plays in improving women’s autonomy (decision-making on health-related issues) and economic freedom, which enabled them to have updated information and access to health care (100, 101). However, in Ethiopia, 40% of women of the reproductive age group have not attended education (6). Acknowledging the gap in education, Ethiopia has introduced a health extension program aimed at providing basic health services for the community along with improving the community’s health-seeking behaviors through continuous health education (102). In addition, formal local structures were established to enhance and facilitate the effort of health extension workers in the community (103). Despite taking these measures, the finding revealed that there is a long way to go in improving the community’s awareness to enhance the uptake of maternity services. Thus, other alternative strategies should be considered to further improve the utilization of maternity services.

Residence was also significantly related to the utilization of ANC service. Women who resided in urban areas had better access to utilization of the ANC services than women who resided in rural areas. The finding was coherent with a study conducted in Ethiopia (104), Nigeria (105), Pakistan (106), Afghanistan (107), Bangladesh (108), and India (109). This is, supposedly, because women who reside in urban areas are expected to have better access to health facilities and information. This, by extension, results in widening the opportunity to receive service from a nearby health facility (110). This is supported by the national survey, which shows that the proportion of women who resided in rural areas and received optimal care declined by half as compared to women who resided in urban areas. In addition, in the Ethiopian context, urban health facilities have better service availability and readiness in the provision of ANC services, which is yet another factor for the notable discrepancy in the utilization of ANC services (111). Acknowledging these notable gaps, the country has put forward measures such as strengthening the health extension programs, firming up the women’s development army, and conducting a series of pregnant women’s conferences, along with implementing continuous quality improvement packages to narrow down the observed discrepancy (20). Overall, the finding implied that a more concerted effort is needed by all the relevant stakeholders to narrow down the inequalities in ANC service utilization.

Type of the region is the other factor associated with the utilization of ANC services, i.e., mothers who resided in city administrations had a better utilization of ANC services, while the opposite was observed among women who resided in the pastoralist region. The result is corroborated by studies conducted in Rwanda (112), Indonesia (113), and India (114). This could be explained by the difference in the accessibility of ANC services, which is usually affected by the availability of roads to the health facility, affordability of healthcare, presence of skilled personnel, and availability of quality care (115, 116). This explanation was also supported by the premise generated from the national survey; women who reside in pastoralist regions barely used the service as set against a woman who resided in city administration (6, 29). On top of this, the utilization of the service was dependent on the husband’s education status, community acceptance, availability of service, early marriage and access to media had a pivotal role in the uptake of the service in the pastoralist regions (54, 111, 117). Thus, the finding imply that coordinated and concerted effort is needed to improve community awareness among the pastoralist community through various means of communication. Additionally, ensuring the availability of essential equipment and adequate manpower should be prioritized as a frontline measure to address the observed inequalities. Furthermore, all these courses of action should be harmonized with Federal and regional health strategies, resources, and interventions to meet national and global targets (118).

Although this study would undoubtedly provide evidence on the determinants of ANC service uptake, it has its own limitation stemming from the type of data used for the analysis, i.e., routine surveillance data. 1) all identified, confirmed, and reported perinatal death through a weekly reporting system were not reviewed and sent via PDRF to the next level, which might introduce potential bias to the study, 2) nearly all deaths were reported and reviewed from public health facilities with limited involvement of private health facilities and the community, and this could affect the representativeness of the study, and 3) a small number of perinatal deaths were captured by the system, which is against national estimates and might compromise the inclusiveness of the study. 4)the study used a cross-sectional approach; therefore, it is difficult to established causality.

## Conclusion

In summary, the utilization of a minimum number of ANC care is low in Ethiopia. Furthermore, the utilization of care among mothers of a deceased perinate is determined by maternal age, educational status, residence, and type of region. Thus, special emphasis should be provided to younger women in improving access to ANC services and addressing barriers to service utilization. Furthermore, enhancing community awareness through different channels of communication along with narrowing down the inequalities in service utilization by providing the required manpower and equipment are some of the mandatory interventions to improve the utilization of ANC service. In addition, detailed qualitative research followed by Delphi excise should be considered to further investigate the effect of the type of region on the frequency of ANC visit to establish a suitable mechanism to improve the uptake of the service in the context of the living condition and culture of pastoralist regions.

## Data availability statement

The datasets presented in this article are not readily available because data cannot be shared publicly because it contains sensitive patient information. Data can be availed by the EPHI data management center after approval by the Research Ethics Committee and Public Health Emergency Management unit. Requests to access the datasets should be directed to info@ndmc.ephi.gov.et.

## Ethics statement

The study was approved by EPHI Scientific and ethical review office (SERO) with Ref. No. EPHI 6_5/437 and permission was obtained from Public Health Emergency Management. We used secondary data obtained from EPHI with no personal identifier information of the participants; henceforth, other ethical measures were inapplicable. Written informed consent for participation was not required for this study in accordance with the national legislation and the institutional requirements.

## Author contributions

NT planned the study and analyzed the literature. MK, MT, and NA coordinated the study. NT, DB, and FW cleaned and analyze data. NT and FW was major contributor in writing the manuscript study. All authors contributed to the article and approved the submitted version.
